# Coincidence of HCV and chronic kidney disease-a systematic review and meta-analysis

**DOI:** 10.1186/s12889-024-20331-0

**Published:** 2024-10-16

**Authors:** Rabia Nawaz, Muhammad Ahmad, Muhammad Saad Raza, Muhammad Rashad, Ayesha Nawaz, Khadija Tabassum, Jalees Ul Hassan, Ammara Ahad, Muhammad Idrees

**Affiliations:** 1 Department of Biological Sciences, Superior University, Lahore, 53700 Pakistan; 2 Division of Molecular Virology, Center of Excellence in Molecular Biology, University of the Punjab, Lahore, Pakistan; 3 University of Veterinary and Animal Sciences, Lahore, Pakistan; 4 Vice chancellor, University of Peshawar, Peshawar, Pakistan

**Keywords:** HCV, Chronic kidney disease, End stage renal disease, Incidence rate ratio, Newcastle ottawa Scale

## Abstract

**Background:**

There are reported studies of Hepatitis C and chronic kidney disease association. However, how this liver virus infection affects the general population’s susceptibility to the onset of the kidney disease is still unknown.

**Methods:**

To determine if a positive anti-HCV serologic status is linked to a greater incidence of chronic kidney disease in the general adult population, a systematic evaluation of the published medical literature since 2015 was conducted. A summary estimate of the relative risk of chronic kidney disease with HCV was produced using a random-effects model. Moreover, stratified analysis and meta-regression were performed.

**Results:**

Twelve studies (*n* = 605858 patients) were filtered and included. Meta-analyses were conducted according to the outcome. Pooling results of longitudinal studies (*n* = 06 studies, *n* = 347120 unique patients) demonstrated an association between positive anti-HCV serologic status and increased incidence of CKD. The summary estimate for adjusted hazard ratio was 1.21 with (95% confidence interval 1.13; 1.29, *P* = 0.001), and between studies heterogeneity was noted (*P* value by Q test < 0.001). In the subset of Asian surveys, the risk of the occurrence of chronic kidney disease linked to HCV was 1.70 (95% confidence interval 1.40; 2.00) without heterogeneity (*P* value by Q test = 0.6).

**Conclusions:**

We found a strong correlation between HCV infection and a higher risk of chronic renal disease in general global population.

## Background

An increasing global public health concern is chronic renal disease. A decrease in glomerular filtration rate and rise in urine albumin excretion are signs of chronic kidney disease (CKD), which affects over 10% of adult members of the general population, according to a number of community-based studies. The prevalence of CKD in the adult population of developed countries is not entirely explained by traditional risk factors for the disease, such as aging, arterial hypertension, metabolic syndrome and diabetes mellitus [1]. Hepatitis C Virus (HCV) infection and glomerulonephritis have been linked in previous studies. We still know very little about the connection between CKD and HCV infection [2]. Glomerular abnormalities have been recorded in up to 85% of individuals with HCV-induced cirrhosis [3].

HCV seroprevalence is about 170 million persons worldwide, and it is one of the significant reasons of chronic liver disorders [4]. HCV can lead to various diseases like Chronic Kidney Disease, Cirrhosis, hepatic or extrahepatic infections, and the hepatic lesions can range from minor ones to hepatocellular cancer, and liver fibrosis [5]. There is strong proof that chronic HCV infection affects tissues and organs other than the liver in significant ways. The activity of the virus on the kidneys is now becoming well known, and a link between HCV infection and CKD has been suggested. Endothelial dysfunction, which is in turn aided by pro-inflammatory cytokines, increased oxidative stress, non-alcoholic steato-hepatitis (NASH) and insulin resistance, can cause renal damage in HCV-positive patients [6].

HCV infection causes mixed cryoglobulinemia and the subsequent onset of membrane proliferative glomerulonephritis, can lead to renal impairment. Therefore, it is believed that effective HCV prevention could reduce kidney impairment [7]. Previous research have investigated how HCV affects CKD risk, but they frequently employed alternative case definitions for both HCV + and impaired kidney activity. For instance, numerous studies have only used antibody testing to define HCV+, which is unable to differentiate between resolved and active, chronic infection. One retrospective study found elevated glomerular filtration rate (eGFR) by at least 10% in veterans who tested positive for HCV antibodies and viremia [8].

Compared to people with adequate kidney function, patients with severe renal insufficiency had a much greater frequency of HCV infection. The difference is especially noticeable in patients receiving hemodialysis, where the prevalence of HCV infection is 13.5% globally vs. 3% in the general population. According to studies, people with ESRD and HCV infection have a 34% higher all-cause mortality rate than patients without the virus [9]. Furthermore, it was conclusively demonstrated that HCV was spread through kidney transplantation (KT) as well as inside of dialysis facilities as a result of a failure of general measures [10]. It was also noted that co-infection with HCV increases the chance of HIV-infected patients developing chronic kidney disease. The kidney disease improving global outcomes work group advises monitoring for creatinine and proteinuria clearance in patients with chronic HCV [1].

We used a systematic review of the literature and a meta-analysis of clinical observational studies to examine the data that is currently available for the relationship among HCV infection and the prevalence of chronic renal disease in the adult population.

## Methods

### Search strategy and data extraction

The present study was conducted in adherence to the Preferred Reporting Items for Systematic Reviews and Meta-Analyses guideline [11]. Appropriate studies were recognized by searching from the following data sources: Medline, Embase, Grateful Med, PubMed and search completed (from 2015 to June 2023) making use of all relevant search phrases. The subsequent search phrases were employed: “Hepatitis C” or “Hepatitis C Virus infection” and “Kidney disease” or “Chronic Kidney disease” (CKD) or “End Stage Renal Disease” (ESRD) and “Glomerulonephritis” or “Low Glomerular Filtration Rate” and “kidney Failure” or “Kidney Impairment” or “Kidney Insufficiency” or “Renal Failure” and “Extrahepatic manifestations” and “HCV” or “HCV antibody positive serologic status” and “Proteinuria” and “Odds Ratio” or “OR” and “Hazard Ratio” or “HR” and “Incidence Rate Ratio” or “IRR”. In order to find more abstracts on the subject, we also manually searched conference papers from significant gastroenterology and hepatology symposia. There were established eligibility and exclusion requirements. Our search was restricted to humans that was written in English-language publications.

### Inclusion criteria

Studies that satisfied the following inclusion requirements were accepted:

(i) They gave unique information from research projects that examined the relationship among CKD and HCV.
(ii) The incidence or prevalence of CKD or ESRD was explicitly identified as the outcome of interest.
(iii) In addition to their 95% confidence intervals, they supplied quantitative risk estimates ( odds ratio [OR], relative risk [RR] or hazard ratio [HR])

We used the most current study in the analysis, if data from the same population were repeated across studies. At the time of enrollment, information regarding HCV serologic status was recorded. We included studies in which HCV infection was identified through the identification of anti-HCV antibodies in serum and/or HCV RNA through nucleic acid testing. The identification of pertinent abstracts, and the subsequent data abstraction from full articles were all carried out independently by two authors, with any disagreements being settled by a third researcher. Study type, year, country, sample count, samples with CKD, follow-up years, sample demographics (such as gender, country, and mean age), follow-up period, and outcome measures were all included in the information that was extracted. If sufficient information on kidney outcomes was present from identified results or given by the study author, then the study was considered eligible and included in the meta-analysis.

### Exclusion criteria

Since letters and reviews were only written as abstracts or were not written in English, they were not included in our analysis. Studies were disregarded if they provided insufficient information about the coincidence among CKD and anti-HCV positivity serologic status (for example, insufficient data on HCV status or kidney outcomes). The studies including the factors and characters beyond the HCV and CKD coincidence, were also exluded, sticking and emphasizing solely on the coincidence of HCV and CKD.

### Outcome measures

We conducted several meta-analyses in accordance with the results. One meta-analysis focused on cohort studies measuring the prevalence of CKD, whereas the other considered longitudinal research evaluating the frequency of CKD.

The main goal was to provide adjusted estimates of the risk (95% CIs) of frequency of chronic renal disease in the majority based on anti-HCV status. The independent impact of anti-HCV-positive status on the prevalence of chronic kidney disease was assessed using multivariate analysis after potential confounders (covariates) such as age, ethnicity, sex, and diabetes mellitus were taken into account. Cohort surveys employed multiple logistic regression analyses, while longitudinal studies employed Cox regression analysis to assess the independent factors influencing the prevalence of chronic kidney disease.

### Quality assessment

A Newcastle Ottawa Scale (NOS) was used to evaluate the study’s quality [12]. The Newcastle-Ottawa scale is a methodologically based rating system that evaluates all the components of an observational epidemiology investigation. One point was added to studies that contained pertinent data that might be connected to the NOS. It was determined that seven items from cross-sectional research and eight from case-control and cohort studies may be connected to the NOS. Thus, studies that were cross-sectional and given scores of 8–10, 6–7, 4–5, or 0–3 were rated as very good, good, satisfactory, or unsatisfactory, in that order. Case-control and cohort studies that received scores of 7–9, 5–6, 4 and 0–3 were also classified as good, very good, satisfactory or unsatisfactory, in that order. Based on the high-quality papers that were submitted, we performed subgroup analyses. Two reviewers worked independently on data extraction and quality scoring, and they combined their findings by consensus. Online access is accessible for the entire quality scoring procedure.

### Data synthesis and analysis

The log odds ratios for case-control and cross-sectional studies and the log hazard ratios for longitudinal studies were divided by the inverse of their variance to provide a pooled effect estimate and its 95% confidence intervals. We chose the approximate value of the impact measure for each trial that had the most confounders taken into account. All random-effects, fixed effects and combined estimates are presented; however, the latter is used and reported when heterogeneity is evident [13]. The heterogeneity was measured using the Cochrane Q test [14]. The percentage of overall variance among trials that may be attributed to diversity rather than chance, or the $\:{I}^{2}$ statistic, was also computed [15]. The lack of heterogeneity is the null hypothesis for this test. By limiting the analysis to research subgroups distinguished by study parameters such as origin country, kidney disease stage, and others, we were able to investigate the cause of heterogeneity. Meta-regression was also used to assess heterogeneity in order to examine the impact of potential and ongoing factors on the target result.

## Results

**Fig. 1:**
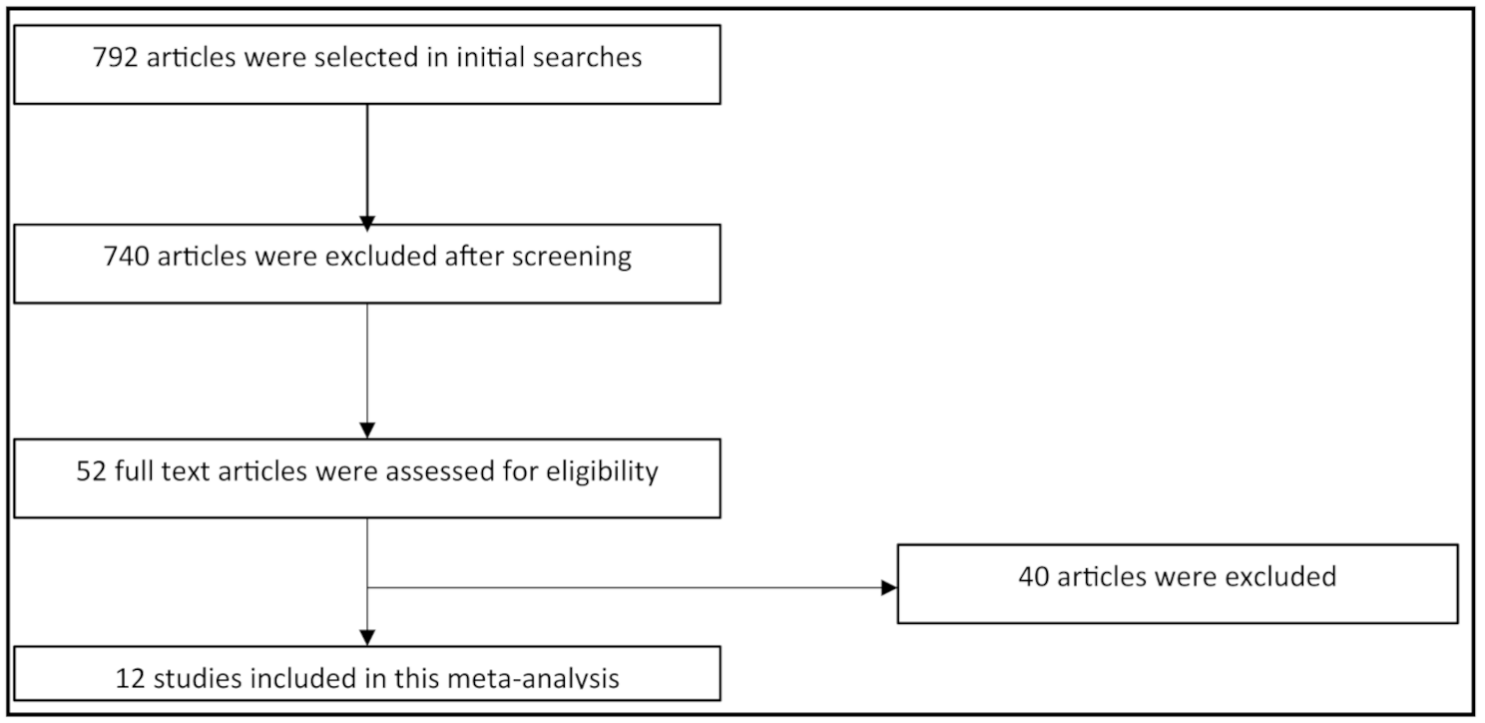
Flow diagram of selected number of studies

After analysing the titles and abstracts of 792 articles found in the initial literature search, 52 were chosen for full-text review. After careful analysis of all the articles, 12 papers which met the eligibility criteria were selected in this study for meta-analysis (Fig. 1).

Included studies reported on 327120 patients from different countries. There were 23344 patients (2 studies) from Taiwan, 322921 patients (2 studies) from USA, 634 from Italy and 221 from Pakistan.The authors have sorted the 52 full-text publications that were evaluated for eligibility, available upon request. A single cohort was included in every meta-analysis, despite certain studies providing data on multiple kidney disease outcomes. Regarding the final inclusion and exclusion of the papers that were reviewed based on the predetermined exclusion and inclusion criteria, there was complete agreement among the reviewers. The most common methods for diagnosing HCV infection were serum anti-HCV antibody testing and PCR based HCV RNA detection.

### Patient and study characteristics

A summary of the key clinical and demographic parameters of the participants in the included studies is provided in Table 1. The patient groups’ mean age varied from 35 to 55 ± 10 years. The distribution of genders showed 45.9 to 70.6% of males. There were six studies: one from Italy, one from Pakistan, two from Taiwan, and two from the USA. For longitudinal research, the quality scores varied from 1 to 4, while for cross-sectional studies, they were between 3 and 4.

**Table 1:** Incidence of CKD in HCV infection

	Tai Shaun Lai	Sara Y.	Haesuk *P*.	Roberto M.	Gantsetseg G.	Izhar Ul H.
Reference Year	2017	2018	2018	2021	2021	2021
Country	Taiwan	USA	USA	Italy	Taiwan	Pakistan
Patients	19,984	153,577	169,344	634	3360	221
Follow-up Years	16.8	10.11	7.0	1.4	12.0	1.0
Males	9804 (49%)	70,527 (45%)	103,149 (60%)	448 (70%)	1715 (51%)	140 (63%)
Anti-HCV-positive cases	591 (3%)	1603 (2%)	56,488 (33%)	60 (9%)	1539 (45%)	68 (30%)
Age	45–55	45–59	48–59	45–65	35–60	25–56
Diabetes mellitus	93 (0.4%)	675 (0.4%)	9231 (5%)	207 (32%)	497 (14%)	0 (0%)
Renal Malfunction	207 (1%)	294 (0.1%)	1455 (0.8%)	40 (6%)	1404 (41%)	221 (100%)
Basis of Renal Malfunction/ CKD detection method	eGFR < 15 ml/min	eGFR < 15 ml/min per 1.73 m^2^	Other Factors like ACEIs, ARBs, congestive heart failure, hypertension etc.	eGFR < 60 ml/min	eGFR < 60 ml/min per 1.73 m^2^	eGFR < 30 ml/min per 1.73 m^2^
Outcome	CKD	CKD	CKD	CKD	CKD	CKD
HCV detection method	HCV antibody and HCV RNA testing	HCV antibody and HCV RNA testing	HCV antibody testing	HCV antibody testing	HCV RNA testing	HCV antibody testing
Adjusted HR (95% CI)	0.92 (0.18, 0.22)	0.64 (0.00, 0.02)	2.42 (0.31, 0.35)	6.02 (0.07, 0.11)	1.08 (0.44, 0.46)	3.02 (0.29, 0.31)

*Tai Shaun Lai et al. [2]: HR adjusted for gender, age, ethnicity, smoking status, educational status. Gantsetseg Gantumur et al. [5]: HR adjusted for gender, age, race, diabetes, baseline GFR, hypertension. Sara Yee Tartof et al. [16]: HR adjusted for gender, age, baseline GFR, diabetes, hypertension, peripheral vascular disease, coronary artery disease, heart failure, chronic obstructive pulmonary disease, alcohol abuse, drug abuse, depression, selected medications. Roberto Minutolo et al. [17]: HR adjusted for gender, age. IZHAR UL HAQ et al. [17]: HR adjusted for gender, age, race, baseline GFR, smoking, hypertension, chronic obstructive pulmonary disease, anemia, dyslipidemia, drug abuse, alcohol abuse. Haesuk Park et al. [19]: HR adjusted for gender, age, diabetes, hypertension, cirrhosis, geographic region, coronary artery disease, urbanization level, enrollee category, number of medical visits in one year before study entry

### Stratified analysis and Meta-regression

Even when the homogeneity criterion was disregarded in certain subgroups, as Tables 2 and 3 illustrate, there was no discernible variation in the pooled aHR across designs. The results of meta-regression showed that the frequency of males had a beneficial impact (*P* = 0.02) on the outcome of interest (adjusted HR of incidence of CKD among HCV-positive patients), as Table 4 lists. In cross-sectional surveys, the prevalence of CKD remained constant across subgroups (Tables 2 and 3).

**Table 2:** A summary metric for the modified effect estimate of incidence among different interest subgroups based on anti-HCV serologic status

Outcome: Chronic kidney fudisease (Incidence), aHR	*N*	Adjusted effect estimate	Q value	I ^2^
Longitudinal studies (All)	6	1.21 (1.13; 1.29)	111,6(*P* < 0.001)	0.89
Longitudinal studies (from USA)	5	1.42 (1.20; 1.64)	65.7 (*P* < 0.001)	0.91
Longitudinal studies (Asian)	3	1.70 (1.40; 2.00)	0.7 (*P* = 0.6)	0.0
Quality score ≥ 3	5	2.06 (1.02; 3.194)	85.3 (*P* < 0.001)	0.74
Quality score ≤ 3	4	1.39 (1.07; 1.79)	21.9 (*P* < 0.001)	0.63

**Table 3:** A summary metric for the modified effect estimate of incidence among different interest subgroups based on anti-HCV serologic status

Outcome: Chronic kidney disease (Prevalence), aOR	*N*	Adjusted effect estimate	Q value	I ^2^
Cross-sectional studies (All)	6	1.53 (1.35; 2.11)	6.55 (*P* = 0.28)	0.13
Cross-sectional studies (USA only)	4	1.19 (0.80; 1.22)	4.56 (*P* = 0.23)	0.37
Cross-sectional studies (Asian)	3	1.002 (0.71; 1.29)	0.44 (*P* = 0.78)	0.02
Cross-sectional studies (with unique cohort)	5	1.27 (1.08; 1.45)	1.63 (*P* = 0.5)	0.17

**Table 4:** Meta-regression analysis: (incidence of CKD)

	Regression coefficient	Standard error	95% CI	Z value	*P* value
Year	**-0.06**	**0.02**	**-0.17; 0.01**	**-1.51**	**0.06**
Size	**-0.00**	**0.01**	**-0.00**, **0.00**	**-1.03**	**0.2**
HCV-positive patients (n)	**-1.29**	**1.13**	**-3.57; 0.78**	**-1.25**	**0.39**
Age	0.02	0.03	-0.02; 0.04	0.64	0.63
Follow-up	0.09	0.05	-0.01; 0.20	1.71	0.07
Diabetes mellitus	1.64	1.08	-0.38; 3.88	1.60	0.12
Male	0.82	0.40	-0.12; 1.71	2.27	0.01
African-American	-0.61	1.06	-2.84; 1.42	-0.65	0.42

### Summary Estimate of Outcome: incidence of CKD

Information on the incidence of CKD among HCV-positive patients was provided by six longitudinal studies (*n* = 327120 unique participants; 60349 HCV-positive and 266771 HCV-negative patients) [2, 5, 16–19] (Table 1). Across all surveys, we discovered a correlation among a positive anti-HCV status and a higher frequency of CKD, adjusted HR with HCV across the surveys, 1.21 with (95% confidence interval 1.13; 1.29, *P* = 0.001), and between studies heterogeneity was noted (*P* value by Q test < 0.001).

### Summary Estimate of Outcome: Prevalance of CKD

Information on the prevalence of CKD among HCV-positive patients was provided by six longitudinal studies (*n* = 259148 unique patients; 58385 HCV-positive and 200763 HCV-negative patients) [7, 8, 19–22]. A few demographic and clinical characteristics of the participants included in trials are displayed in Table 5. Across all surveys, we did not find a correlation among positive anti-HCV serologic status and a higher prevalance of CKD.

**Table 5:** Prevalence of CKD in HCV infection

	Varun Saxena	Hwang J	Park H	Michel Jadoul	Yoshimasa T	Eugenia Wong
Reference Year	2016	2016	2017	2018	2021	2021
Country	UK	Taiwan	USA	USA	Japan	USA
Patients	1789	19,574	225,792	946	207	10,430
Males	1140 (63%)	10,044 (51%)	137,231 (60%)	548 (57%)	92 (44%)	6547 (62%)
Anti-HCV-positive cases	950 (53%)	9787 (50%)	56,448 (25%)	141 (14%)	143 (69%)	112 (1%)
Age	55–65	43–67	35–60	40–63	58–70	57–63
Diabetes mellitus	415 (23%)	19,574 (100%)	36,739 (16%)	368 (38%)	36 (17%)	2185 (20%)
Renal Dysfunction	186 (10%)	1004 (5%)	1404 (0.6%)	321 (34%)	27 (13%)	1626 (15%)
Outcome	CKD	CKD	CKD	CKD	CKD	CKD
Adjusted OR (95% CI)	0.9 (0.88, 0.92)	1.00 (0.43, 0.57)	0.89 (0.24, 0.26)	1.00 (0.12, 0.14)	0.98 (0.67, 0.71)	1.00 (0.00, 0.02)

*Varun Saxena et al. [20]: OR adjusted for gender, age, ethnicity, smoking status, educational status, hypertension, diabetes. Hwang J et al. [21]: OR adjusted for gender, age, race, hypertension, diabetes. Park H et al. [19]: OR adjusted for gender, age, HBsAg, systolic blood pressure, and fasting plasma glucose. Michel Jadoul et al. [22]: OR adjusted for gender, age, body mass index, educational status, albumin level, cholesterol level, hemoglobin level, hypertension, and diabetes. Yoshimasa T et al. [7]: OR adjusted for gender, age, alcohol drinking, hypertension, body mass index, smoking, fasting glucose, cholesterol, triglyceride, uric acid, waist-to-height ratio, creatinine. Eugenia Wong et al. [8]: OR adjusted for gender, age, ethnicity, smoking status, diabetes, educational status, hypertension and diabetes

#### Incidence of CKD in HCV infection

Values shown as HCV-positive/HCV-negative individuals.

#### Prevalence of CKD in HCV infection

values shown as HCV-positive/HCV-negative individuals.

## Discussion

The earliest reports of HCV infection involving the kidneys were made twenty years ago; nevertheless, there is conflicting and little information regarding the relationship between low eGFR in the adult population and HCV [23]. We have conducted a meta-analysis and compiled the scientific data regarding adult exposure to HCV infection and the likelihood of developing chronic renal disease. This meta-analysis (twelve studies, *n* = 605858 patients) confirms the interaction between positive serologic status for HCV and CKD, aHR being 1.21 (95% CI 1.13; 1.29, *P* = 0.001), after results from six studies.

Longitudinal studies showed a coincidence between HCV and incidence of CKD (aHR, 1.42, 95% CI 1.20; 1.64, *P* = 0.001) in the adult population. Even though our stratified analysis revealed uniform results in the subset of research from Asia, the presence of significant heterogeneity definitely hindered more decisive conclusions. Thus, it is clear that HCV infection and the development of CKD are closely related in Asian nations; further research from these and other continents is needed.

It is clear that not all of the sources of heterogeneity we have seen could be captured by our subgroup study using meta-regression. Numerous pieces of data support the idea that HCV has a negative impact on CKD. There is a huge correlation among a positive anti-HCV serologic status and accelerated progression of CKD, as shown by cohort studies conducted in patients with HCV and HIV disorders [24], diabetes, patients with biopsy-proven glomerulonephritis, and cirrhosis patients who finished IFN therapy [25]. Regardless of blood transfusion, individuals with chronic kidney illness had a considerably greater seroprevalence of anti-HCV antibody before they reached end-stage renal disease compared to the general population [26].

In numerous studies, there was no significant correlation found between the prevalence of chronic renal disease and anti-HCV positive status. One possible explanation for the disparity between cross-sectional and longitudinal research is the absence of a suitable follow-up [27]. Our meta-regression analysis revealed that the influence of HCV on the incidence of CKD was stronger in longitudinal studies with older populations and longer follow-up periods. The stratified analysis revealed a steady correlation between the prevalence of chronic renal disease and anti-HCV positive serologic status. In comparison with other meta-analysis conducted by Fabrizi F et al. our results of meta regression analysis are more significant, which show strong relationship between HCV and CKD [1].

Our meta-analysis’s conclusions are constrained in a few ways. First of all, a large number of research used a cross-sectional design, which relies heavily on prevalence ratios and precludes the ability to draw conclusions about causality. Limiting analysis to cross-sectional studies revealed no correlation between the prevalence of CKD and positive anti-HCV serologic status; this could be explained by the increased mortality rate among HCV-positive patients. There is then some evidence to suggest that the study design may have an impact on the findings. Second, results were largely consistent when analyses were limited to studies with high quality scores; nevertheless, residual confounding (confounding that persists after adjustment) probably exists because not all of the retrieved studies provided complete information on all of the confounders. For instance, information on HCV RNA, socioeconomic status, follow-up visit compliance, and substance abuse all significant potential confounders was lacking. In a new Taiwanese survey, the interaction between HCV and CKD remained significant (aHR 1.08; 95% CI 0.44; 0.46, *P* = 0.0009), even after HCV-positive individuals without traditional CKD risk factors (hypertension, diabetes, coronary artery disease, cirrhosis and hyperlipidemia) were eliminated. Ultimately, not all of the sources of heterogeneity we have seen could be captured by our subgroup analysis using meta-regression. One significant cause of study heterogeneity may be misclassification and measurement error in the evaluation of proteinuria or chronic renal disease. We concentrated on the random-effects estimates for our interpretation because of the substantial estimates of heterogeneity. The effect of small studies is given more weights by the random effects model, which could lead to bias in the estimation. It is noteworthy that in certain analyses, there exists a significant disparity between the random-effects and fixed effect estimates. This discrepancy could perhaps be attributed to variations in confounder adjustment.

## Conclusion

This meta-analysis shows that, among adults in general, there is a significant connection between HCV infection and CKD. To lessen the variety we have seen, future research should be predicated on a standard diagnosis of chronic renal disease. The connection between HCV infection and a higher risk of renal disease may be due to confounding variables such as exposure to nephrotoxic medications and injectable drug use. Until further studies proving otherwise, individuals infected with HCV should be considered to be more vulnerable to CKD, even in the absence of traditional risk factors for renal disease.

## Data Availability

All data is included in this manuscript.

## Abbreviations

CKD: Chronic Kidney Disease
HCV: Hepatitis C Virus
NASH: Non-alcoholic Steato-hepatitis
eGFR: Estimated glomerulus filtration rate
KT: Kidney transplantation
ESRD: End stage renal disease
OR: Odds ratio
HR: Hazard ratio
IDD: Incidence Rate Ratio
NOS: Newcastle Ottawa Scale
